# The Protective Effect of Health Literacy on Reducing College Students' Stress and Anxiety During the COVID-19 Pandemic

**DOI:** 10.3389/fpsyt.2022.878884

**Published:** 2022-05-19

**Authors:** Yuting Ying, Chunxia Jing, Fan Zhang

**Affiliations:** ^1^Department of Biostatistics and Epidemiology, School of Public Health, Sun Yat–sen University, Guangzhou, China; ^2^Department of Epidemiology, School of Medicine, Jinan University, Guangzhou, China; ^3^Department of Public Health and Preventive Medicine, School of Medicine, Jinan University, Guangzhou, China

**Keywords:** health literacy, college students, anxiety, stress, COVID-19

## Abstract

**Background:**

The coronavirus (COVID-19) pandemic threatens people's health and well–being all around the world, resulting in increased stress and anxiety. Existing literature has found health literacy has a protective effect on health, and the study has taken a closer look at the effects of health literacy on perceived stress and anxiety among Chinese college students.

**Methods:**

With structural questionnaires, a cross–sectional survey was conducted to collect the responses of 1,251 participants from different universities in Hubei and Guangdong, China. Participants' health literacy and perceived stress and anxiety symptoms were evaluated.

**Results:**

Only 11.83% of the participants reported sufficient health literacy. Compared with college students from Hubei and Guangdong with a major in medicine showed a higher percentage of having sufficient literacy. Moreover, having sufficient health literacy showed a protective effect in reducing the risk of stress (OR = 0.14, 95%CI= 0.01–0.04; *p* < 0.001) and anxiety (OR = 0.02, 95%CI = 0–0.61; *p* < 0.001).

**Conclusion:**

Health literacy was found to have a protective effect in reducing anxiety and stress among college students. This effect has remained among students from different majors and locations. However, it is noteworthy that the overall level of health literacy is relatively low among college students, particularly among those from Hubei Province or with non–medical majors. Therefore, more effort should be put into developing health education programs promoting health literacy and mental health on campus.

## Introduction

Since late December 2019 in Wuhan, China (1, 2), the coronavirus disease (COVID-19) has spread rapidly in China and worldwide. COVID-19 has cast a detrimental effect on people's physical and emotional wellbeing (3). People reported heightened degrees of loneliness, despair, anxiety, and stress related to the pandemic and its corresponding social distancing policies (4). Furthermore, the psychological consequences of COVID-19 have been discovered in a variety of populations, including patients with COVID-19, healthcare providers, and the elderly (5). College students may also be vulnerable to mental health problems considering the various challenges they face while moving from adolescence to young adulthood, including the collective stress and financial strain in the context of a pandemic (6). Therefore, it is critical to address the impact of the COVID-19 pandemic on the protective factors that affect college student's overall mental health. Particularly, greater mindfulness and social support, which were found to be a protective factor for psychological health (7).

Among the COVID-19–related psychological manifestations, one of the most common issues under the impact of the pandemic was the high prevalence of anxiety (8, 9). The increase in cases of COVID-19 infection and mortality has sparked widespread concern and anxiety (10). In previous research investigating psychological reactions during the outbreak of COVID-19 in China, Wang et al. found that more than half of the subjects felt moderate to severe psychological distress (e.g., avoidance, intrusion, and hyperarousal) toward the pandemic, while one–third suffered from moderate or severe anxiety (11). In China, women suffered from a greater psychological impact of the pandemic and exhibited higher levels of stress, anxiety, and depression (12, 13). For college students, the uncertainty of enrollment, online participation, and examination during the pandemic may negatively affect their mental health. A previous study revealed that college students reported higher levels of stress, anxiety, and depression (13).

Emerging research has recognized health literacy (HL) as a critical factor in promoting health, quality of life, and well–being. HL was defined as “the degree to which individuals can obtain, process, and understand health information and services they have to make appropriate health decisions” (14). Individuals with limited HL often lack knowledge about healthy lifestyles and the causes of diseases (15). Meanwhile, people with sufficient HL reported better quality of life and mental health (16), and fewer symptoms of depression and anxiety (17, 18). To explain this effect, it is possible that higher HL could encourage people to adopt healthy behaviors (19), form healthy lifestyles (20), and reduce their uncertainties when facing the pandemic (21). However, it has remained unclear how HL may influence college students' stress and anxiety in relation to the COVID-19 pandemic. Evidence on the association between HL and psychological disturbances is limited. In addition, the levels of HL among college students varied across age, gender, and field of study (22, 23). Moreover, limited HL was associated with a higher risk of obesity and smoking (24).

Little research was conducted on the HL of college students, and the existing evidence suggested that the overall level of HL remained limited (25). College students play an important role in the economic growth and development of a society (6). The majority of college students, although physically mature, are still mentally unstable and immature. In undergoing various physical and psychological changes while facing great uncertainties and stress, adolescence is the peak stage of the onset of mental disorders (26, 27). The student's needs for prompt and effective treatment for mental disorders have exceeded the available resources, resulting in a large unmet need for mental illness treatment among college students (28, 29). Therefore, understanding the situation of college students' mental health and addressing the protective factors is pivotal for developing health–promoting services on campus.

According to a national assessment of Chinese residents' HL, 27.43% of people aged 15 to 24 had appropriate HL in 2020 (30). Despite this, limited HL may contribute to a range of adverse health outcomes for college students (31, 32). According to Drissi et al., strengthening college students' HL is critical in reducing the risk of mental health problems (33). Compared with other majors, medical students usually have a higher level of HL (22). However, medical students also showed a higher risk for mental illnesses than non–medical students (34). Particularly, depressive symptoms, anxiety, and stress symptoms are more common among medical students (35). In addition, the context of the college may also play a role. Hubei province has been the hardest hit by COVID-19, with 188 people affected physically and psychologically. Meanwhile, Guangdong province faced numerous obstacles during the epidemic due to its large population movements. As a result, college students from Hubei and Guangdong may be at higher risk of mental illness during the pandemic. Hence, in the current study, we have recruited and compared the students from these two provinces.

To sum up, the current study investigated how HL affects anxiety and stress among college students in China during the COVID-19 pandemic to have a better understanding of college students' HL and its impact on mental health. We hypothesized that those with limited HL would be more likely to show anxiety symptoms under the impact of the pandemic.

## Materials and Methods

### Study Design and Participants

A cross–sectional study was conducted from December 2020 to February 2021. With a convenience sampling from six universities in Hubei and Guangdong, China, we have collected data with an online platform “Wenjuanxing” (Changsha Haoxing Information Technology Co., Ltd., China) (36), which was widely used in previous research (37). Before data collection, a pilot survey was conducted to test the feasibility of the measurements, and minor adjustments to the questionnaire were made accordingly. The link of the survey was shared with 1,380 college students *via* snowball sampling, resulting in 1,275 responses. The minimal required sample size was calculated by taking the reference of sample size calculation in the “Chinese Citizens' Health Literacy Survey” as follows: [N=U^2^π(1–π) ÷δ^2^× deff]. In this formula, U was 1.96, the design effect (deff) was set to 3.0, and the allowable error δ was.05. A total sample size of 1,130 was obtained with a 10% possible rate of invalid response. In total, 1,275 responses were collected.

Eligible participants were 18 years or older and were studying in universities in Hubei and Guangdong provinces, mainland China. This survey was entirely voluntary for participants. To avoid missing data, each IP address can only access the link once, and anonymous responses can only be submitted if all of the questions have been answered. The questionnaire did not include any personal information or sensitive content. In addition, attention check questions, such as “1 + 1 =?”, were included in the survey to test whether the participants were paying attention while answering questions. Response times of <180 s will be considered as invalid data, and the responses were deemed invalid if the participants choose the same option for over 80% of consecutive items in the survey. After the screening, 98.20% of the responses (*N* = 1,251) were included in the analysis.

### Measurements

Demographic information was collected, including participants' age, gender, grade, major, ethic, and family income (per month).

#### Health Literacy

The Chinese Citizen health literacy survey questionnaire, designed by the China Health Education Center, was used to measure participants' HL (38). The questionnaire included three dimensions, namely, health belief and knowledge, health behavior, and health skill literacy (39). Twenty items were listed, in which wrong answers and “Do not know” were scored 0 points, and the correct answer was scored 1. The total score of HL was calculated by adding the scores of all items. Participants who had a total HL (HL) score of 16 or above would be coded as having “Sufficient HL (score ≥16),” and those with a score lower than 16 were coded as having “limited HL (score <16).” Cronbach's alpha in this study was 82.

#### Mental Health

The Generalized Anxiety Disorder Questionnaire (GAD−7) (40–42) was used to estimate an individual's anxiety symptoms. It is commonly used in clinical practice and research because of its diagnostic reliability and efficiency (43). The questionnaire includes 7 items, assessing participants' anxiety symptoms such as feeling nervous, anxious, inability to stop worrying, excessive worries, etc. (44). GAD−7 uses a 4–point Likert scale in scoring, with 0 indicating “Not at all,” and 3 indicating “almost every day.” The total score ranges from 0**–**20, with a score of ≥5, ≥ 10, and ≥15 representing mild, moderate, and severe anxiety, respectively (45). Cronbach's alpha in this study is 98.

The Chinese version of the perceived stress scale (CPSS) (46) was used to measure perceived stress among college students. The scale includes 14 items, each having five responses, with 0 indicating “never” and 4 indicating “always.” The total score ranged from 0**–**56. A total score between 15 and 28 was deemed moderate stress, a total score ranging from 29**–**42 was reckoned as strong stress, and a score of above 42 was considered as intense stress. The CPSS demonstrates strong reliability and validity in a Chinese population (47), with a Cronbach's alpha of.90 in this study.

### Data Analysis

Data analyses were performed using STATA software (Version 15.0 for Mac). To address the group differences, a *T*–test and one–way ANOVA were used. We used multivariate logistic regression to explore the effect of HL on anxiety and stress. We also explored the main effect of major on HL. Demographic variables, including age, gender, major, province, ethnicity, region, and family income, were controlled in the adjusted models.

## Results

A total of 1,251 participants were incorporated into the analysis, with a valid response rate of 98.20%. The descriptive results of the participants were shown in Table 1. Overall, the mean age was 21.4 years (SD = 2.52), with 47.8% male and 32.5% medical students. Approximately 60% of the participants were from Guangdong, with 72.5% of them being undergraduates. More than 50% of participants are placed in urban areas, and 26% have a family income of <5,000 yuan per month. The average HL score was 7.6, and only 11.83% of participants showed sufficient HL (with a total HL score ≥16). Participants with sufficient HL are more likely to be female (16.39%), older, medical students (27.83%), with higher grades, and with better family income from Guangdong province. The mean score of CPSS and GAD−7 was 27.3 (SD = 9.16) and 13.1 (SD = 7.02), respectively, (see Table 2). As seen in Table 3, HL was negatively correlated with anxiety level (*p* < 0.05) and stress level (*p* < 0.05).

**Table 1:** Participants' characteristics (*N* = 1,251).

**Demographics**	**Mean or Frequencies (%)**	***p*** **–value**
**Age, years**	21.36 ± 2.51	<0.001
**Gender**		
Male	598 (47.8)	<0.001
Female	653 (52.2)	
**Province**		
Hubei	514 (41.1)	<0.001
Guangdong	737 (58.9)	
**Major**		
Liberal arts	373 (29.8)	<0.001
Science	472 (37.7)	
Medicine	406 (32.5)	
**Grade**		
Undergraduate	907 (72.5)	<0.001
Postgraduate	310 (24.8)	
Doctor	34 (2.7)	
**Ethic, Han**	1076 (86.0)	<0.001
**Region, Urban**	637 (50.9)	<0.001
**Family Income (per m)**		
<4999	325 (26.0)	<0.001
5000–6999	484 (38.7)	
≥7000	442 (35.3)	
**Sufficient Health literacy** **Diagnosed with anxiety**	148 (11.83)	<0.001
Weak	109 (8.71)	<0.001
Moderate	135 (10.79)	
Strong	745 (59.55)	
**Diagnosed with stress**		
Weak	152 (12.15)	<0.001
Moderate	557 (44.52)	
Strong	449 (35.89)	
Intense	93 (7.43)	
Total	1251 (100.0)	

**Table 2:** Mean scores of the sample from the Chinese version of the Perceived Stress Scale (CPSS) and Generalized Anxiety Disorder questionnaire (GAD−7).

	**M+N–MAX**	**Mean (SD)**
Perceived Stress Scale	0~56	27.3 ± 9.16
Anxiety	0~21	13.1 ± 7.02

**Table 3:** Correlation matrix of the relationship between the study variables.

**Variables**	**1**	**2**	**3**	**4**	**5**	**6**	**7**	**8**	**9**
1. Health Literacy.	1								
2. Anxiety Level	**–**0.429^*^	1							
3. Stress Level	−0.216^*^	0.175^*^	1						
4. Gender	0.147^*^	−0.131^*^	−0.020^*^	1					
5. Age	0.394^*^	−0.269^*^	−0.092^*^	0.080^*^	1				
6. Ethic	−0.126^*^	0.096^*^	−0.005	−0.025	−0.071^*^	1			
7. Region	−0.097^*^	0.073^*^	0.034	−0.018	−0.001	0.028	1		
8. Major	0.302^*^	−0.162^*^	−0.085^*^	0.071^*^	0.200^*^	−0.040	−0.023	1	
9. Grade	0.410^*^	−0.247^*^	−0.096^*^	0.071^*^	0.815^*^	−0.085^*^	−0.048	0.219^*^	1

* *p < 0.05*.

Sufficient HL is a protective effect on stress (see Table 4). A participant with limited HL is more likely to perceive greater stress (*p* < 0.001). Participants with moderate stress levels had an Risk Ratio (RR) of.42 (95% CI = 0.27–0.65, *p* < 0.001). Meanwhile, those with strong stress levels had an RR of.17 (95% CI = 0.10–0.29, *p* < 0.001), and those with intense stress levels had an RR of.03 (95% CI = 0.00–0.21, *p* < 0.001). We further adjusted the data to reflect age, sex, province, and major, with which the results remained similar (moderate stress level: RR = 0.43, 95%CI = 24–0.77; strong stress level: RR = 0.22, 95%CI = 12–0.43; high intensity stress level: RR = 0.50, 95%CI = 0–0.45; *p* for trend < 0.001).

**Table 4:** The association between health literacy and stress level.

**Health literacy**	***p*** **for trend**	**Stress level**							
		**Weak**		**Moderate**		**Strong**		**High intensity**	
		**RR (95%CI)**	***p***	**RR (95%CI)**	***p***	**RR (95%CI)**	***p***	**RR (95%CI)**	***p***
**Model 1**									
Limited	<0.001	Reference		0.42 (0.27–0.65)	< 0.001	0.17 (0.10–0.29)	< 0.001	0.03 (0.00–0.21)	<0.001
Sufficient									
**Model 2**									
Limited	<0.001	Reference		0.37 (0.22–0.61)	<0.001	0.17 (0.09–0.30)	<0.001	0.03 (0.00–0.26)	0.001
Sufficient									
**Model 3**									
Limited	<0.001	Reference		0.43 (0.24–0.77)	.005	0.22 (0.12–0.43)	<0.001	0.05 (0.00–0.45)	0.007
Sufficient									

*Model 1, Unadjusted; Model 2, Adjusted for age and gender; Model 3, Adjusted for age, sex, province, major, grade, region, incomes*.

Sufficient HL was associated with reduced anxiety (see Table 5). Participants with limited HL were more likely to experience a higher level of anxiety (*p* for trend <0.001). The RR of participants with moderate anxiety level was.28 (95% CI = 0.15–0.52, *p* < 0.001), and that with strong anxiety level was 14 (95% CI = 0.01–0.04, *p* < 0.001). We further adjusted for age, sex, province, major, grade, and family income, and the results remained similar (moderate anxiety level: RR = 0.13, 95%CI = 02–0.76; strong anxiety level: RR = 0.02, 95%CI = 0.00–0.61; *p* for trend < 0.001).

**Table 5:** The association between health literacy and anxiety level.

**Health literacy**	***p*** **for trend**	**Anxiety level**							
		**Normal**		**Weak**		**Moderate**		**Strong**	
		**RR (95%CI)**	***p***	**RR (95%CI)**	***p***	**RR (95%CI)**	***p***	**RR (95%CI)**	***p***
**Model** 1									
Limited	<0.001	Reference		2.94 (1.85–4.68)	<0.001	0.28 (0.15–0.52)	<0.001	0.14 (0.01–0.04)	<0.001
Sufficient									
**Model 2**									
Limited	<0.001	Reference		2.20 (1.31–3.69)	<0.001	0.31 (0.16–0.61)	<0.001	0.02 (0.01–0.06)	0.001
Sufficient									
**Model 3**									
Limited	<0.001	Reference		5.63 (1.25–25.42)	0.025	0.13 (0.02–0.76)	<0.001	0.02 (0.00–0.61)	0.025
Sufficient									

*Model 1, Unadjusted; Model 2, Adjusted for age and gender; Model 3, Adjusted for age, sex, province, major, grade, region, incomes*.

## Discussion

Health literacy research is still an emerging research area, and the influence of HL on college student's mental health has not been well understood (48). This study measured HL, perceived stress, and anxiety among Chinese college students. Compared to medical students, HL among non–medical students was lower. However, HL had an overall positive effect on reducing anxiety symptoms and stress among college students.

Health literacy has attracted growing research attention. Developing HL skills early in life can have beneficial effects on education and academic performance, which may have long–term benefits. Sufficient HL could contribute to improved health outcomes, better health care, and decision–making in health–related situations (49, 50). Additionally, it would also help college students to better adapt to their campus life and future workplace, thus positively impacting public health (51, 52).

Based on the results of this study, only 11.83% reported sufficient HL, and the rate was even lower among non–medical students. As mentioned by an action plan to improve HL developed by the United States Center for Disease Control (US CDC) is commendable for: “(1) developing and disseminating accurate, accessible, and actionable health and safety information; (2) integrating clear communication and HL into public health planning, funding, and, policy development, research and evaluation; and (3) the inclusion of accurate, standards–based and developmentally appropriate health and science information and curricula in educational settings from childcare to college level” (53). Considering our findings, further health education should be developed on campus with actionable health information, to improve students' health literacy levels. Further health education should also be integrated into the curriculum of general education to ensure that students from different majors could all access to and apply health information in their daily life.

With the data of 1,251 participants in this survey, about 60% of college students showed anxiety symptoms, and 43.43% perceived strong or intense stress. Moreover, limited HL was associated with a higher level of perceived stress (54), and this is consistent with a prior survey that indicated limited HL was negatively associated with anxiety (17, 55). Individuals with sufficient HL usually better understand health information, and thus know better about how to cope with the infection risk. Therefore, they have a means to maintain or reduced anxiety or stress. In contrast, people with limited HL may have difficulty in obtaining related health knowledge, and experience increased stress and anxiety level (56). A previous study showed that mental disorders like anxiety and perceived stress could negatively affect college students' HL (57), and our cross–sectional findings provided more evidence supporting this negative association without determining the specific causal relationship.

The current study has focused on college student's mental health and the protective effect of HL. College students are the cornerstone of society's future. Their future life and career would be compromised by lacking sufficient HL. Our findings have underscored that it is necessary to develop health education to cultivate students' HL, which would further benefit their mental well–being. To better meet the public health demands under the impact of the pandemic, HL is more important than ever before. HL would enable individuals to take better care of their physical and mental health, particularly during the COVID-19 pandemic, and enhancing HL among college students would have long–term benefits for public health (53).

### Limitations

Despite the insights from the findings, the limitations have to be acknowledged. The current study was conducted by cross–sectional data. As mentioned above, longitudinal studies are warranted to better understand the causal relationship between HL and mental health. Online questionnaires were used to obtain data from college students in two provinces in mainland China. Thus, the sample may not be enough to be a representative of the overall college students. Hence, cautions should be taken when generalizing the findings into other populations. To ensure the generalizability of our findings, more research with a larger sample size is required. Furthermore, participants' experiences during the pandemic were important to mental health. However, our pilot survey indicated that only <1% of participants have reported “knowing or having close contact with people infected with COVID-19.” In addition, no sick or suspected cases were included in this study, implying that we may have a selection bias in our sample. Moreover, we have adopted a culture–specific measurement of HL, which may lead to inconsistent findings with a different instrument. Lastly, more cross–cultural comparisons should be done in the future.

Despite the limitations, this study sheds light on the mental health of college students. The results will be insightful for developing health education programs or mental health services for both medical and non–medical college students. Adequate support should be provided to increase college students' HL to reduce the risk of anxiety symptoms and stress. HL is a national and international priority, and improving the asset of HL would indicate more opportunities for health and lifelong learning. It is relevant to a whole–school, whole–community, and whole–child approach, and it also involves healthcare professionals, institutions, and systems at different levels of the society (58). The current study provides evidence about an updated profile of HL among Chinese college students and its effects on anxiety symptoms and stress during the pandemics.

## Data Availability Statement

The original contributions presented in the study are included in the article/Supplementary Material, further inquiries can be directed to the corresponding authors.

## Ethics Statement

The studies involving human participants were reviewed and approved by Human Research Ethics Committee of Jinan University. The patients/participants provided their written informed consent to participate in this study.

## Author Contributions

YY contributed to collecting data, performed the data analysis, and wrote the paper. FZ and CJ conceived and designed the whole paper. All authors contributed to the article and approved the submitted version.

## Conflict of Interest

The authors declare that the research was conducted in the absence of any commercial or financial relationships that could be construed as a potential conflict of interest.

## Publisher's Note

All claims expressed in this article are solely those of the authors and do not necessarily represent those of their affiliated organizations, or those of the publisher, the editors and the reviewers. Any product that may be evaluated in this article, or claim that may be made by its manufacturer, is not guaranteed or endorsed by the publisher.
