# Supplementary material for: The Protective Effect of Health Literacy on Reducing College Students' Stress and Anxiety During the COVID-19 Pandemic
Source: Front Psychiatry. 2022 May 19;13:878884. doi: 10.3389/fpsyt.2022.878884 (PMC9161275; doi:10.3389/fpsyt.2022.878884)
Supplement: Supplementary file 1 [file Table_1.docx]

**eTable1. Mean scores of the sample on the CPSS, GAD-7**

|  | **M+N-MAX** | **Mean (SD)** |
| --- | --- | --- |
| Perceived Stress Scale | 0~56 | 27.3± 9.16 |
| Anxiety | 0~21 | 13.1 ±7.02 |
